# Melt-Cast Films Significantly Enhance Triamcinolone Acetonide Delivery to the Deeper Ocular Tissues

**DOI:** 10.3390/pharmaceutics11040158

**Published:** 2019-04-02

**Authors:** Akshaya Tatke, Narendar Dudhipala, Karthik Yadav Janga, Bhavik Soneta, Bharathi Avula, Soumyajit Majumdar

**Affiliations:** 1 Department of Pharmaceutics and Drug Delivery, School of Pharmacy, The University of Mississippi, Oxford, MS 38677, USA; aatatke@go.olemiss.edu (A.T.); ndudhipa@olemiss.edu (N.D.); kjanga@go.olemiss.edu (K.Y.J.); bksoneta@go.olemiss.edu (B.S.); 2 Research Institute of Pharmaceutical Sciences, The University of Mississippi, Oxford, MS 38677, USA; bavula@olemiss.edu; 3 National Center for Natural Products Research, The University of Mississippi, Oxford, MS 38677, USA

**Keywords:** Triamcinolone acetonide, PEO, Soluplus^®^, in vitro release, melt-cast, permeability, ocular distribution

## Abstract

Delivering an effective drug load to the posterior section of the ocular tissues, while using a non-invasive technique, has always been a challenge. In this regard, the goal of the present study was to develop sustained release triamcinolone acetonide (TA) loaded polymeric matrix films for ocular delivery. The TA-films were prepared in two different polymer matrices, with drug loadings of 10% and 20% *w*/*w*, and they were evaluated for ocular distribution in vivo in a conscious rabbit model. A 4% *w*/*v* TA suspension (TA-C) was used as a control for in vitro and in vivo studies. The TA-films, prepared with melt-cast technology, used polyethylene oxide (PEO) and Soluplus^®^ as the polymer matrix. The films were evaluated with respect to assay, content uniformity, excipient interaction, and permeability across isolated rabbit sclera. The distribution of TA in the ocular tissues, post topical administration, was determined in New Zealand male albino rabbits as a function of dose, and was compared against TA-C. The assay of the 10% and 20% *w*/*w* film was in the range from 70–79% and 92–94% for the Soluplus^®^ and PEO films, respectively, and content uniformity was in the range of 95–103% for both the films. The assay of the TA from Soluplus^®^ films was less compared with the PEO films and showed an interaction with TA, as revealed by Differential Scanning Calorimetry (DSC). Hence, Soluplus^®^ films were not selected for further studies. No interaction was observed between the drug and PEO polymer matrix. The enhancement of trans-scleral flux and permeability of TA was about 1.16 and 1.33-folds, respectively, from the 10% *w*/*w* PEO and 3.5 and 2.12-folds, respectively, from the 20% *w*/*w* PEO films, as compared with TA-C formulations. The in vivo studies demonstrate that significantly higher TA levels were observed in the anterior and posterior segments of the eye at the end of 6h with the PEO films. Therefore, the PEO based polymeric films were able to deliver TA into the back of the eye efficiently and for prolonged periods.

## 1. Introduction

Ophthalmic drug delivery is a challenging task because of the complex physiology and structure of the eye. Topical ophthalmic drug delivery is the most preferred method in the treatment of the ocular diseases due to their effortless and painless application to the eye. However, there are many physiological and anatomical restraints, which include tear dilution, nasolacrimal drainage, loss due to blinking, low formulation volume, or low residence time and tissue barriers, such as cornea and conjunctiva, which extensively inhibits the drug permeation [1,2].

Triamcinolone acetonide (TA), which is a steroidal anti-inflammatory drug, falls under Biological classification system (BCS) class 4 category, which means that the drug exhibits poor solubility and permeation characteristics [3]. TA is mainly used in the treatment of posterior ocular diseases, such as inflammation, posterior uveitis, and diabetic macular edema. The topical TA solution or suspension eye drops are effective for the disorders in the anterior segment of the eye, but they are not as such effective for the posterior segment [4,5,6]. The current practice is to administer TA suspension as an intravitreal injection [7,8]. However, this procedure is associated with side-effects, such as increased ocular pressure, retinal hemorrhage, and endophthalmitis [9,10].

Recently, many researchers have investigated various alternate topical ophthalmic drug delivery formulations [11]. The development of formulations with a lipid base, like lipid nanoparticles [12], liposomes [6], nano micellar drops [13], or the addition of drug penetration enhancers in the formulation have been approaches evaluated for the delivery of a wide range of drug classes [14]. Besides these, ophthalmic formulation strategies have been studied to attain a controlled release pattern of the drug. Some of the strategies include the use of in situ gels [15], inserts [16], contact lenses [17], collagen shields [18], etc. With the added advantage of extensive residence time and the adhesion of the formulation to the ocular tissues, these formulations could show a higher bioavailability of the drug with minimal drug loss. 

Ocular inserts are polymeric solid or semi-solid, thin, sterile films, which are placed into the conjunctival sac or cul-de-sac. With the convenience of direct application, these formulations display advantages, such as direct tissue contact for extended periods of time along with sustained release [19]. Due to presence of polymers, the release pattern of TA from the ocular inserts can be modeled in accordance with the type of drug as well as the target delivery. The ideal mechanisms of the drug release from these inserts are diffusion, osmosis, or bioerosion [20]. The inserts are prepared by utilizing water soluble polymeric materials that can be molded into flat inserts, without using solvents, by the melt-cast technique [21,22]. Polymeric films of TA for enhancement of posterior and anterior ocular segment delivery have not been reported until now. Hence, we aimed to develop and characterize sustained release TA inserts for ocular tissues. 

Polyethylene oxide N-10 (PEO) is a nonionic hydrophilic, low viscosity, and safe polymer, which, in the presence of water, turns into a gel with high swelling capacity. The melting range of PEO is between 63 to 68 °C, which is highly suitable for the melt-cast formulation process and for achieving smooth flexible films, as, at that temperature range, the polymer also exhibits thermoplastic behaviour that allows for uniform drug distribution [23,24]. Soluplus^®^ is a grafted copolymer, which is a combination of hydrophilic and hydrophobic elements. Hence, the polymer can be utilized as a film matrix as well as a solubility enhancer. The low glass transition temperature of the Soluplus^®^ i.e., 70 °C makes it an ideal candidate for melt-cast film base [25].

Therefore, the current study focuses on formulating TA in the form of ocular inserts, with PEO and Soluplus^®^ as polymers, while using the melt-cast method and evaluating their in vitro and in vivo performance. 

## 2. Materials and Methods

### 2.1. Chemicals

PEO (PEO N-10) and Soluplus^®^ were kind gift samples from Dow chemical company (Midland, MI, USA) and Badische Anilin- und Soda-Fabrik (BASF) (Research Triangle Park, NC, USA), respectively. TA, high-performance liquid chromatography (HPLC)-grade solvents, optima grade solvents for Liquid Chromatography Mass spectroscopy (LCMS), and other analytical grade chemicals were obtained from Fisher Scientific (Hampton, NH, USA).

### 2.2. Animals and Animal Ocular Tissues

Whole eye globes were procured from Pel-Freez Biologicals (Rogers, AR, USA). For the in vivo studies, male albino New Zealand rabbits were ordered from Envigo (Indianapolis, IN, USA). The in vivo drug distribution studies were accomplished in accordance with the University of Mississippi Institutional Animal Care and Use Committee approved protocol (17-018).

### 2.3. Preparation of TA-Films

The drug-polymeric film was prepared by melt-cast procedure. Two drug concentrations, 10% and 20% *w*/*w*, were selected to prepare films utilizing the PEO and Soluplus^®^ as the film matrix. Initially, both the drug and polymer base were separately weighed. The drug was added on to the polymer by geometric dilution and the mixture was triturated after each addition. The final mixture was added into the 13 mm die that was placed over a brass plate. The whole system was placed over the hot plate with the temperature of the plate set at around 70 to 80 °C. To get disc films, the mixture in the die cavity was compressed with a punch. As the brass plate heats, and the temperature reaches the glass transition temperature, the polymer begins to melt, forming a translucent film. The brass plate was then removed from heat and kept aside for air cooling for the formulation to form a thin flat disc. Later, the disc was carefully cut to the dimension of 4 mm × 2 mm (0.2–0.4 mm thickness) segments, such that the weight of the pieces was approx. 8 mg with the load of 0.8 mg and 1.6 mg of the drug, respectively, for 10% and 20% *w*/*w* drug loads. These films were used for all further studies. The films that were prepared were kept in a closed container at room temperature to check for any physical or chemical instability over a period of time [22].

TA control (TA-C) suspension, 4% *w*/*v*, was prepared in accordance with the composition of marketed intravitreal injection Triesence^®^. Briefly, TA was suspended in a suitable volume of deionized water, while using sodium CMC as a suspending agent, to achieve the same concentration as the 20% *w*/*w* polymeric film.

### 2.4. HPLC Chromatographic Conditions

The drug concentrations in all in vitrosamples were analyzed using HPLC, employing Waters 600 pump controller and Waters 717 plus autosampler. The drug concentration was determined by Waters 2487 ultraviolet (UV) dual absorbance detector at detection wavelength (λ_max_) of 254 nm and peak areas were integrated by 3395 Agilent integrator. The column used for analyzing the samples was C_18_ Luna^®^ 4.6mm × 250 mm and mobile phase consisted of 1:1 isocratic solution of acetonitrile (ACN) and water. All of the experiments were carried out in triplicates.

### 2.5. Characterization of TA-Films

#### 2.5.1. Differential Scanning Calorimetry (DSC)

Prior to formulating the films via melt-cast technique, differential scanning calorimetry analysis was used to determine the interaction between the drug and the polymers that were used. DSC spectrum of pure TA, PEO and Soluplus^®^ were obtained while using a DSC 250 (TA instruments, New Castle, DE, USA). Approximately 5 mg of the samples were sealed in different T0 aluminium pans and they were scanned from 0 to 300 °C with constant nitrogen purging at a heating rate of 20 °C/min. Thermograms were plotted on the basis of change in the heat flow as compared to sealed blank aluminium pan taken as reference.

#### 2.5.2. Drug Content Uniformity and Assay

Four sections of the film, with theoretical drug contents of 0.8 or 1.6 mg for 10% and 20% films, respectively, were isolated from the different zones of the film and were dissolved in a 1:1 ratio of methanol and DMSO to determine the drug quantity in the different sections. To measure the content in the formulation, the whole films were dissolved in 1:1 ratio of methanol and DMSO to get a clear solution. The solution was then appropriately diluted in methanol and drug concentration was analyzed by the HPLC method, as discussed earlier. The films were analyzed for both the drug content and content uniformity over the period of two months.

#### 2.5.3. In vitro Release Study

TA release from the TA-films was investigated to understand the release rate and the drug diffusion pattern from the films into the release media. Prior to the release study, the saturation solubility of the drug was analysed in different media. Based on the solubility results, Dulbecco’s phosphate-buffered saline (DPBS) (pH 7.4), with 5% hydroxyl propyl β cyclodextrin (HP-β-CD), was selected as the receiver medium. The films were placed at the base of dry glass vials. A brass mesh (#10) was placed on top of the film with a magnetic stirrer above the mesh. The whole system was placed under a constant temperature of 34 ± 0.2 °C with continuous magnetic stirring up to the end of study. Ten mL of the receiver medium was added to each vial and approximately 1 mL aliquots was withdrawn at the predetermined time points and replaced with an equal quantity of receiver medium. The concentration of the drug in the aliquot was analysed using HPLC method, as discussed above.

#### 2.5.4. In Vitro Tissue Permeation Study

The rabbit eye tissues were employed for performing the in vitro tissue permeation studies. The eyes that were acquired from Pel-Freez Biologicals were shipped in ice cold Hanks Balanced Salt Solution. The tissues were immediately separated after receipt and washed with ice cold DPBS solution (pH 7.4). As in the in vitro release study, the receiver medium was DPBS (pH 7.4) with 5% HP-β-CD. The temperature of the diffusion cells was maintained at 34 ± 0.2 °C with continuous circulating water and the system was running under constant magnetic stirring. The scleral tissues were mounted on the Valia–Chien diffusion cells with the epidermis of the sclera facing towards donor chamber [26]. Formulations used for examining the rate of permeation were TA-C, 10% and 20% TA films with about 0.2–0.4 mm thickness and 4 mm × 2 mm dimensions. Fifty microlitres of DPBS was added along with 8 mg of the film in the donor chamber, to attain similar conditions as in vivo. Aliquots (600 µL) were withdrawn from the receiver chamber at different time points and they were replaced with same amount of DPBS (pH 7.4) with 5% HP-β-CD, to maintain the sink conditions. The drug contents in the samples were analysed in multiples of three, using the HPLC method.

Cumulative amount of the drug permeated (*M_n_*), trans-scleral steady state flux (J) and trans-scleral tissue permeability were calculated. The equation used for calculating cumulative amount of the drug was:

(1) $$M_{n}=V_{r}C_{r\left(n\right)}+\sum_{x=1}^{x=n}V_{s\left(x-1\right)}C_{r\left(x-1\right)}$$

where, *n* was sampling time point; *V_r_* and *V_S_* were the volume of the receiver chamber (mL) and the volume of the sample collected at the nth time point (in mL), respectively, and Cr(*n*) is the concentration of the drug in the receiver chamber medium at nth time point (µg/mL).

The steady state flux of the drug was determined based on the slope that was obtained from the graph of cumulative amount of TA transported versus time. The steady state flux of TA was calculated while using the equation:

(2) Flux(J)=(dM/dt)/A

where, *M* was the cumulative amount of drug transported and *A* was the surface area of the cornea (0.636 cm^2^).

The trans-scleral permeability of TA was calculated by the following equation:

(3) $$Permeability\;\left(Papp\right)=\frac{Steady\;state\;flux}{Donor\;concentration}$$

#### 2.5.5. In Vivo Tear Kinetics and Drug Distribution Studies

Male New Zealand albino rabbits (weighing 2.0 to 3.0 kg) were used to study the in vivo drug distribution and tear kinetics. Prior to the study, the animals were quarantined for seven days in the animal facility, to allow for acclimatization, with complete access to water and food. For the study, the animals were divided into three groups and each group received different formulations: 10% TA-film, 20% TA-film, or 4% *w*/*v* TA-C. For TA-C, 40 µL of the formulation was added to the left eye of the animal—the other eye was untreated. For the films, 4 mm × 2 mm segments of 10% and 20% TA films were placed accurately in the cul de sac of the left eye of the animals. The eyes were checked for signs of redness or irritation after administration of the formulations. After drug administration, approximately 5 µL of tear samples were collected from each animal every hour until six hours using a micropipette. Around 10 min. prior to the last time point of tear collection i.e., 6 h, the animals were anaesthetized with an intramuscular injection of a combination of ketamine and xylazine. After the animals were anaesthetized, pentobarbital was administered via the ear vein to euthanize them. Subsequently, both the test and the non-treated eyes were carefully separated and washed individually with ice-cold IPBS (isotonic phosphate-buffered saline) solution. The ocular tissues were cautiously isolated and stored at -80 °C until further analysis. The tear samples were extracted in methanol and were later analyzed for drug content using HPLC-UV. Total area under the curve (AUC) and half-life (*t*_1/2_) was calculated. The formula that was used for calculation of AUC was

(4) $$AUC=\sum \left[\frac{\left(C_{1}+C_{2}\right)}{2}\times \left(t_{2}-t_{1}\right)\right]$$

where *C_1_* and *C_2_* are drug concentration in tear at time *t*_1_ and *t*_2_, respectively, and the formula for calculating *t*_1/2_ was

(5) t12=0.693Ke

where *K*_e_ is calculated as −2.303 × the slope of the plot time v/s log concentration.

TA was extracted from the ocular tissues, including aqueous humor (AH), vitreous humor (VH), cornea, sclera, iris-ciliary bodies (IC) and retina-choroid (RC), and the concentration was quantified using UPLC-triple quadrupole (TQ)-MS system.

##### Sample Extraction from the Ocular Tissues

TA was extracted from the ocular tissues by protein precipitation technique. To about 200 µL of AH and 500 µL of VH, 100 µL of 1 µg/mL of prednisolone (PR) as internal standard (IS) was added and vortexed. The tissue proteins were precipitated using 200 µL and 500 µL of ice-cold methanol for AH and VH, respectively, and then vortexed again. The weights of the isolated solid ocular tissues: cornea, IC, RC, and the sclera were noted, and the tissues were transferred to separate the eppendorf tubes, and they were further cut into small pieces to ensure the effective extraction of TA from the tissues. The IS (1 µg/mL) of 100 µL was then added to each of the tissue sample and let stand. After that, 1mL of ice-cold methanol with 0.1% formic acid was added as a protein precipitating solvent. To extract the drug into the solvent, all of the samples were vortexed thrice for about 30 s, let stand at room temperature for 15 min, and then sonicated for about 5 min. The samples were then centrifuged for 15 min. at 13,000 rpm and the supernatant was collected and stored at −80 °C until further analysis.

##### Bioanalytical Analysis

The TA concentration in all of the ocular tissues was quantified using Ultra Performance Liquid Chromatography system coupled with a triple quadrupole mass spectrometer (UPLC-TQ-MS) (Waters, Milford, MA, USA). The peaks of both the drug and the IS were quantified with respect to the specific mass to charge (*m*/*z*) values (*m*/*z* 435 and 361 for TA and PR, respectively). Two microliters of the sample were eluted through BEH (ethylene bridged hybrid) C18 (100 mm × 2.1 m, 1.7 µm) Acquity UPLC^®^ column and separated using an isocratic mobile phase consisting of ACN with 0.1% formic acid and water with 0.1% formic acid in the ratio of 98:2 respectively. The method showed limits of detection (LOD) and quantification (LOQ) of 0.1 ng/mL for TA and IS respectively. All of the the instrument functions were operated and managed by Mass Lynx software (version 4.1, Waters, Milford, MA, USA).

##### Statistical Analysis

The results for all of the analyses are represented by their mean and SD values. The difference in drug concentration or permeability coefficient for both films was checked for their statistical significance as compared to TA-C formulation. To analyze the statistical difference and to calculate the p-value with the confidence interval of 95%, a one-way analysis of variance (ANOVA) along with Tukey’s post hoc test (version 5.00; GraphPad Prism Software, San Diego, California, USA) was utilized.

## 3. Results and Discussion

### 3.1. Preparation of TA Polymeric Films

TA polymeric films were formulated by evenly blending and distributing the drug in the melted polymer matrix, PEO and Soluplus^®^, by melt-cast method. The drug concentrations in these polymeric films were 10 and 20% *w*/*w*. The melt cast process coverts the drug to its amorphous form, or molecular dispersion state, which in turn increases the solubility of the drug. Increased solubility increases the permeability of the drug across the membrane, thus increasing the bioavailability of the drug to the target tissues [22]. Additionally, the inserts localize high drug concentrations into the matrix without the need for any organic solvent. The modifications in the matrix using different polymers/grades or combinations of two or more polymers provides the necessary flexibility that is needed to accommodate various drug classes, including both hydrophobic and hydrophilic drugs, with tailored drug release patterns in the physiological tissues.

### 3.2. Drug Uniformity and Assay

The assay (%) values for both 10% and 20% *w*/*w* TA PEO based films were more than 92% (Table 1). However, the assay values for TA films that were formulated using Soluplus^®^ as the film matrix were significantly lower than the PEO based films (72.5–78.8%). This difference in the assay results may be due to the incomplete entrapment of the molecule in the Soluplus^®^ polymer. Irrespective of the assay, content uniformity in both PEO and Soluplus^®^ based films were 95.3 ± 7.5 and 102.5 ± 5.2;102 ± 11.4 and 92.4 ± 9.0% for 10% and 20% drug loads, respectively. This indicates the complete and uniform drug distribution into the polymer matrix during the formulation process.

To evaluate the storage stability, the films were placed in vials and then stored at room temperature. The films did not show any physical change in the time span of two months. The stability data, Table 1, also reveals no significant variations in the films characteristics i.e., assay and drug uniformity during the test period. However, variation was observed (102.0 ± 11.4 and 88.9 ± 3.8 % at day 1 and day 60, respectively) in the content uniformity of 10% *w*/*w* Soluplus^®^ film. Thus, the assay and drug uniformity outcomes indicate that the PEO based films have superior stability and TA loading capabilities.

### 3.3. Differential Scanning Calorimetry (DSC)

**Figure 1:**
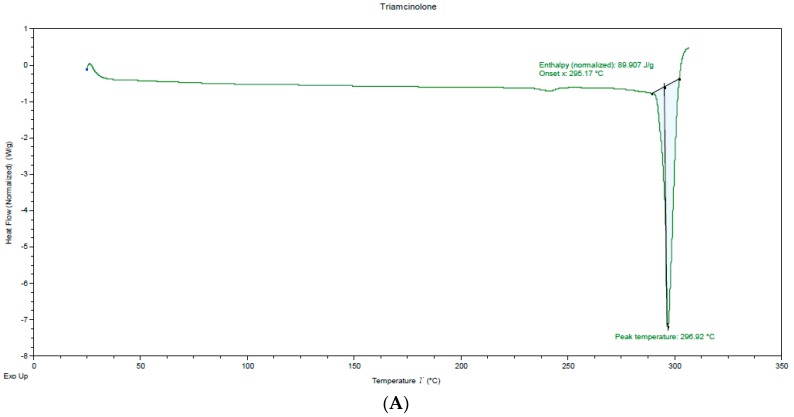

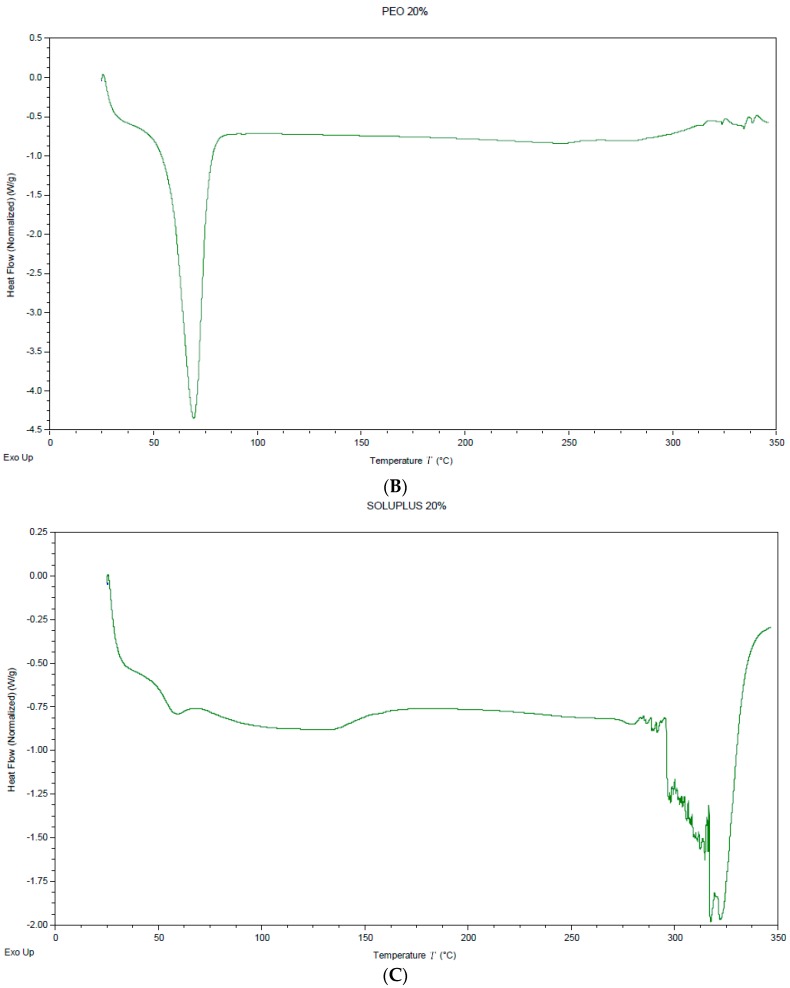
Differential Scanning Calorimetry (DSC) thermograms of pure drug (**A**), PEO film (**B**), and Soluplus^®^ film (**C**).

Figure 1 illustrates the DSC thermograms of TA and the physical mixture of TA with the polymers. The thermogram for TA shows the melting point of the drug at around 290 °C, as indicated by the occurrence of a major endothermic event. In the thermogram of TA with PEO, there is a depression at around 70 °C that corresponds to melting point of the polymer matrix, followed by constant heat flow up to 320 °C, which suggests that TA is either solubilized by PEO or exists in the amorphous form in the film. However, in the thermogram of the films that were prepared with Soluplus^®^, a thermal event is observed at around the melting point of the TA suggesting the incompatibility or low solubility of TA in the polymer—which eventually affects the assay of overall formulation.

### 3.4. In Vitro Release Study

**Figure 2:**
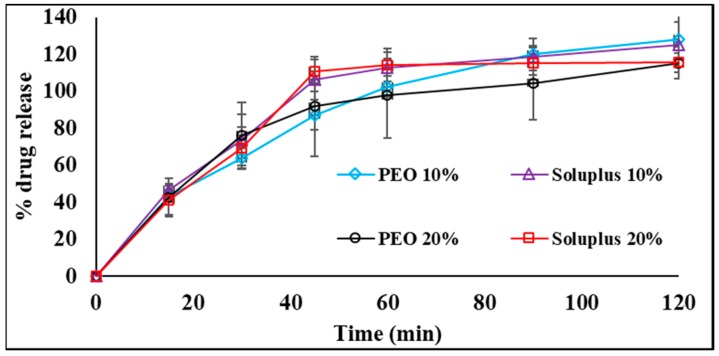
Drug release profiles of triamcinolone acetonide from 10% and 20% *w*/*w* PEO and Soluplus^®^ films (mean ± SD, *n* = 3). *,^#^ indicates that the permeability and flux of PEO TA-films were statistically significant when compared to control at *p* < 0.05.

The saturation solubility studies demonstrate that DPBS with 5% HP-β-CD solution was ideal as the receiver medium as the drug solubility was found to be highest in that medium. This receiver medium was utilized for both in vitro release and in vitro tissue permeation studies. Figure 2 presents the release profile of TA from both the 10% and 20% *w*/*w* loaded films. Hundred percent release of the drug can be observed from the Soluplus^®^ films (both 10 and 20%) well within 45 min. However, the release of TA from PEO reaches 100% post one hour. This might be due to swelling of the PEO in the presence of water, which causes the delay in the release of the drug [23]. Based on the film characteristics, in vitrodrug release profiles and drug interaction studies, PEO films were selected for further in vitro tissue permeation and in vivo studies.

### 3.5. In vitro Tissue Permeation 

**Figure 3:**
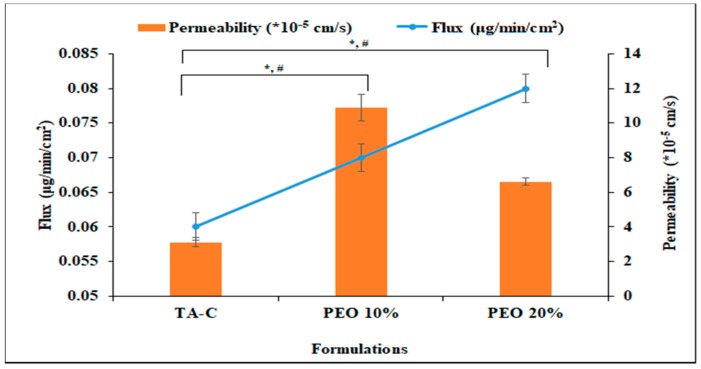
In vitro drug permeation of triamcinolone acetonide from 10% and 20% *w*/*w* PEO and films and polyethylene oxide (TA-C) formulation (mean ± SD, *n* = 3).

Trans-scleral permeation studies were carried out with both 10 and 20% *w*/*w* TA loaded PEO films and using TA-C as the control. Figure 3 presents the results. The concentration of the drug in the TA-C formulation was maintained as similar to that of the drug content in the 20% PEO film. The permeability of TA across the sclera from TA-C, 10% PEO film, and 20% PEO film was found to be 3.5 ± 0.2, 10.9 ± 0.7, and 6.6 ± 0.2 × 10^−5^ cm/s, respectively. The flux from the TA-C, 10% PEO, and 20% PEO films were calculated to be 0.06 ± 0.005, 0.07 ± 0.005, and 0.08 ± 0.001 μg/min/cm^2^, respectively. Trans-scleral TA flux did not change significantly, but the permeability increased 3.5 and 2.12-folds from the 10% and 20% PEO films, respectively, when compared to that from the TA-C formulation.

This observation can be explained by the fact that PEO, being a hydrophilic polymer, dissolves the drug, as evident from the DSC thermograms, and this solubilized TA penetrates across the cornea and increases the permeability. Whereas, in the TA-C, more drug was in solid state, which resulted in low penetration across the cornea. Hence, the permeability and flux of TA from the films was superior to TA-C [22].

### 3.6. In vivo Tear Kinetics and Ocular Distribution Studies

PEO based TA films (both 10% and 20% *w*/*w*) and TA-C formulations were selected for the in vivo studies. Films of size 4 mm × 2 mm were placed in the cul-de-sac (left eye) of New Zealand albino rabbits. For the TA-C formulation, 40 µL of the formulation was instilled into the left eye cul-de-sac. The films gel within 5–10 min on contact with the tear fluid and it sticks to the scleral section in the cul-de-sac without interfering with vision, which is consistent with our earlier report [23]. The drug concentration in tear was analysed every hour up to 6 h and Table 2 presents the data. Since the tear kinetics of TA from the melt-cast film were being studied for the first time, a 6 h investigation period was considered to be appropriate. Longer study durations, under this experimental design, could lead to undetectable drug levels in the deeper ocular tissues. Thus, the experiment was designed to obtain maximum information with respect to the effectiveness of the melt-cast films over the suspension. The results show low/no TA levels in the tear of the control group rabbits that were treated with TA suspension, while rabbits treated with films showed significant TA levels in the tear at 6h. The highest concentration of TA in tear was observed at the first hour from the TA-C formulation (42.7 ± 19.2 µg/mL), while for the PEO films, the highest concentration of the drug was observed in the third hour: 21.9 ± 6.1 µg/mL and 38.1 ± 15.1 µg/mL for 10% and 20% films, respectively. The half-life (T_1/2_) of TA in the tear film was calculated to be 1.7 ± 0.3 h, 3.7 ± 2.6 h, and 7.2 ± 1.1 h for the TA-C and 10% and 20% films, respectively. The total area under the curve (AUC) for the 10% and 20% films was approx. 1.35 and three-folds higher, respectively, than AUC for TA-C. This high AUC is because of the formation of a polymeric gel in tear, followed by slow release of the drug. The tear kinetics demonstrates the improved retention and controlled release of TA into the tear from the matrix film [27,28]. Further, this study design may somewhat underestimate the drug penetrating into the ocular tissues due to sampling loss. However, the half-life of topically administered formulations is less than 10 minutes, thus the zero-time point sampling could be the only procedure that could lead to drug loss that could affect the ocular kinetics.

In our earlier investigations, we reported the ocular safety and biocompatibility of the melt cast films. Furthermore, no redness and irritation in the eyes of rabbits were seen during the in vivo experiment [23]. In the current study, no signs of irritation were observed with both the 10% and 20% TA loaded films.

**Figure 4:**
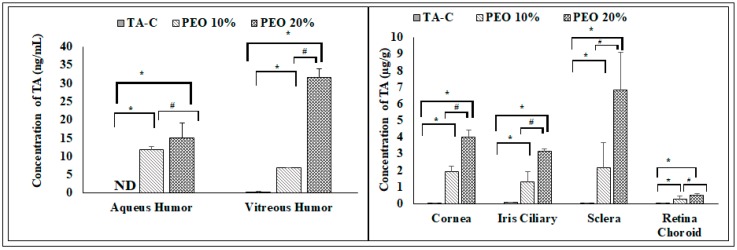
In vivo ocular distribution of triamcinolone acetonide from 10% and 20% *w*/*w* PEO films and TA-C in ocular tissues (mean ± SD, *n* = 3). *, ^#^ indicates *p* < 0.05 statistically significant of 20% PEO TA-films when compared to TA-C and 10% PEO TA-films, respectively. ND—not detected.

The TA concentration in the ocular tissues from TA-C, and from the film formulations, 6 h post dosing, as depicted in Figure 4. The drug concentration in the AH and VH was determined to be 11.7 ± 0.9 ng/mL and 6.9 ± 0.01 ng/mL, respectively, for 10% PEO film formulation and 15.1 ± 3.6 ng/mL and 31.6 ± 2.2 ng/mL, respectively, for 20% PEO films. However, the concentration for TA-C in AH posts 6 h dosing was below the limit of detection, while in VH the concentration was 0.36 ± 0.09 ng/mL.

In the case of the solid tissues, the TA concentration was found to be 0.011 ± 0.001 µg/g in the cornea, 0.076 ± 0.006 µg/g in the IC, 0.022 ± 0.01 µg/g in the RC, and 0.019 ± 0.03 µg/g in the sclera from the TA-C formulation. From the film formulation, the concentration of TA in cornea, IC, RC, and sclera were found out to be 1.9 ± 0.3 µg/g, 1.3 ± 0.6 µg/g, 0.2 ± 0.1 µg/g, and 2.1 ± 1.4 µg/g, respectively, for the 10% PEO films and 3.9 ± 0.4 µg/g, 3.1 ± 0.1µg/g, 0.5 ± 0.1 µg/g, and 6.8 ± 2.2 µg/g, respectively, for the 20% PEO films.

The concentration of the TA in the ocular tissues that were generated from the films was significantly higher when compared to that observed with the TA-C formulation, 6h after dosing. Additionally, the concentration of the drug from the 20% PEO films in the VH, cornea, IC, and sclera were significantly higher than those that were obtained with the 10% PEO films (*p* > 95%). In the posterior segment ocular tissues, as compared to TA-C formulation, the amounts of the drug detected in the VH, RC, and sclera were about 19.3, 12.2, and 114.7 times greater, respectively, from the 10% PEO film formulation and 87.9, 23.1, and 359.4 times, respectively, from the 20% films. Similarly, the TA levels in cornea and IC were 172.7 and 17.1 folds higher from 10% PEO film, 354.5 and 40.7 folds higher from the 20% PEO films, respectively. Further, 20% PEO films showed about 4.5, 1.8, and 3.1 folds higher TA levels in VH, RC, and sclera when compared to the 10% PEO films. 

As seen from the release studies, PEO, being a water-soluble polymer, formed a gel in the presence of the tear and it resided as a gel formulation in the cul-de-sac. Due to higher drug partitioning into the tissues that are next to cul-de-sac, a high amount of TA was found in corneal and scleral tissues. Thus, high drug levels were observed in the posterior section of the ocular tissues, even after 6 h post administration indicating the utility of PEO as film matrix base for topical ocular inserts [21,22,29,30].

Previously, the ocular delivery of TA loaded nanostructured lipid carrier (TA-NLCs) formulations were developed and their performance was tested in mice, using fluorescence intensity measurement with confocal microscope by Araújo et al., [5] after topical administration. Nile red (NR) was used as a fluorescent lipid marker. From the results, TA-NLC treated eyes showed strong fluorescence in first 8 min. and it was also detectable at 160 min., on the surface of the anterior segment. However, fluorescence was not detected in the case of eyes that were treated with NR solution, either through the systemic, corneal, or non-corneal pathways in the 160 min. experiment duration. However, the study did not present any data on the TA concentrations that were accomplished in the ocular tissues. 

In another study, Altamirano-Vallejo et al. evaluated the vitreoretinal delivery of TA topical liposomes [6]. Ocular disposition studies were carried out in rabbits and TA concentrations were determined at the end of 12 h, 1, 7, and 14 days in retina and VH, following treatment with topical TA liposome formulation every 2 h, six times, during 14 days. The authors state that the highest levels of TA were observed at the end of 12 h in retina (0.252 µg/g) and VH (36 ng/mL), and it eventually decreased at the end of 14th day following a first order elimination kinetics. However, the dosing regimen of the study was not clearly understood. In the current study, high levels of TA were obtained from the 10% and 20% TA films with about 6.9 ± 0.01 ng/mL and 31.6 ± 2.2 ng/mL in the VH, and 0.2 ± 0.1 µg/g and 0.5 ± 0.1 µg/g in the RC, respectively, at the end of 6 h, following a single topical dose. 

Recently, we reported the in situ gel of TA loaded solid lipid nanoparticles (TA-SLN-IG) for topical delivery for the back of the eye [31]. The ocular disposition studies revealed that, in AH and VH, the TA levels that were obtained from the TA-SLN-IG formulation, 6 h post dosing, were 4.2 and 3.3-folds greater than the 10%, and 6.4 and 1.4-folds higher from the 20% films. In contrast, the TA levels in the cornea, IC, RC, and sclera were 3.7-, 6.7-, 2.7-, and 6-6-folds and 7.6-, 16.1-, 5.1- 20.6-folds greater from the 10% and 20% films, respectively, when compared to TA-SLN-IG. Therefore, the results strongly suggest that the TA films could be an alternative approach for topical delivery of TA as ophthalmic formulations.

## 4. Conclusions

TA polymeric films were successfully developed with high drug content and good uniformity and stability. The formulations needed a minimum use of excipients. With the PEO films, the matrix swells in the presence of the tear fluid and behaves as gels. The trans-scleral permeability of the drug from the PEO films showed high permeation, with better flux results when compared to the TA-C. Tear kinetics from the film dosage form showed high TA concentrations at the third hour post administration and with sustained release as compared with TA-C. In vivo drug distribution data from the PEO films showed the dose related drug concentration. The amount of the drug in the ocular tissues from the film formulations were found to be significantly higher after six-hour post formulation administration, suggesting a sustained release drug delivery platform. Thus, the formulation of TA as PEO based melt-cast films present an efficient approach for delivery to the anterior and posterior segment ocular tissues.

## Figures and Tables

**Table 1:** Assay and drug uniformity content of triamcinolone acetonide 10% and 20% *w*/*w* polyethylene oxide (PEO) and Soluplus^®^ melt-cast film formulations (mean ± SD, *n* = 3).

Formulation	Time (Days)	Assay	Drug Content Uniformity
TP10	1	92.40 ± 0.26	95.39 ± 7.54
	30	92.03 ± 0.64	92.59 ± 0.60
	60	92.31 ± 1.18	89.45 ± 3.94
TP20	1	93.94 ± 3.07	102.54 ± 5.21
	30	92.29 ± 3.55	94.15 ± 1.83
	60	92.40 ± 1.06	93.87 ± 0.21
TS10	1	72.56 ± 0.10	102.01 ± 11.45
	30	72.14 ± 2.43	90.70 ± 1.38
	60	70.76 ± 1.83	88.90 ± 3.87
TS20	1	78.82 ± 0.10	92.42 ± 9.09
	30	77.03 ± 1.85	90.05 ± 0.80
	60	75.87 ± 2.65	89.09 ± 2.48

TP and TS indicates melt-cast films of triamcinolone acetonide (TA) with PEO and TA with Soluplus^®^ respectively and 10 and 20 indicates the total drug content (in percent) in the whole film.

**Table 2:** In vivo tear kinetics of triamcinolone acetonide from TA-C and 10 and 20% PEO formulations (mean ± SD, *n* = 3).

Formulation	Concentration of Triamcinolone Acetonide in Tear at Different Time Points (µg/mL)						AUC (µg•h/mL)	T_1/2_ (h)
	0 h	1 h	2 h	3 h	4 h	6 h		
**TA-C**	**0**	42.77 ± 19.29	5.90 ± 3.53	5.09 ± 0.58	1.35 ± 0.22	1.03 ± 0.17	26.51 ± 8.04	1.72 ± 0.3
10% PEO film	0	10.70 ± 1.85	15.18 ± 2.60 *	21.99 ± 6.15*	17.33 ± 4.64 *	12.05 ± 2.51 *	86.84 ± 12.41 *	3.77 ± 2.6*
20% PEO film	0	12.76 ± 2.51	21.97 ± 6.86 *	38.14 ± 15.19 *, ^#^	32.02 ± 7.51 *, ^#^	28.12 ± 5.45 *, ^#^	148.68 ± 13.76 *, ^#^	7.28 ± 1.19 *, ^#^

* indicates statistically significant values for both the PEO films compared to TA-C (*p* < 0.01); ^#^ indicates statistically significant at *p* < 0.05 of 20% PEO film when compared with 10% PEO film.

## References

[1] Duvvuri S., Majumdar S., Mitra A.K. (2003). Drug delivery to the retina: Challenges and opportunities. Expert. Opin. Biol. Ther..

[2] Gaudana R., Ananthula H.K., Parenky A., Mitra A.K. (2010). Ocular Drug Delivery. AAPS J..

[3] Jermak C.M., Dellacroce J.T., Heffez J., Peyman G.A. (2007). Triamcinolone acetonide in ocular therapeutics. Surv. Ophthalmol..

[4] Clares B., Gallardo V., Medina M.M., Ruiz M. (2009). Multilamellar liposomes of triamcinolone acetonide: Preparation, stability, and characterization. J. Liposome Res..

[5] Araújo J., Nikolic S., Egea M.A., Souto E.B., Garcia M.L. (2011). Nanostructured lipid carriers for triamcinolone acetonide delivery to the posterior segment of the eye. Colloids. Surf. B Biointerface.

[6] Altamirano-Vallejo J.C., Navarro-Partida J., Gonzalez-Dela R.A., Hsiao J.H., Olguín-Gutierrez J.S., Gonzalez-Villegas A.C. (2018). Characterization and pharmacokinetics of triamcinolone acetonide-loaded liposomes topical formulations for vitreoretinal drug delivery. J. Ocul. Pharmacol. Ther..

[7] Morrison V.L., Koh H.J., Cheng L., Bessho K., Davidson M.C., Freeman W.R. (2006). Intravitreal toxicity of the kenalog vehicle (benzyl alcohol) in rabbits. Retina.

[8] Vasumathy V., Ramasamy K. (2006). Intravitreal injection of triamcinolone acetonide for diabetic macular edema: Principles and practice. Ind. J. Opthamol..

[9] Wingate R.J., Beaumont P.E. (1999). Intravitreal triamcinolone and elevated intraocular pressure. Aust. N. Z. J. Ophthalmol..

[10] Jonas J.B., Kreissig I., Söfker A., Degenring R.F. (2003). Intravitreal injection of triamcinolone for diffuse diabetic macular edema. Arch. Ophthalmol..

[11] Janoria K.G., Gunda S., Boddu S.H.S., Mitra A.K. (2007). Novel approaches to retinal drug delivery. Expert Opin. Drug. Deliv..

[12] Gan L., Wang J., Jiang M., Bartlett H., Ouyang D., Eperjesi F., Liu J., Gan Y. (2013). Recent advances in topical ophthalmic drug delivery with lipid-based nanocarriers. Drug Discov. Today.

[13] Chodankar R., Dev A. (2017). Formulation and characterization of triamcinolone acetonide emulgel. World J. Pharm. Pharm. Sci..

[14] Kaur I.P., Smitha R. (2002). Penetration enhancers and ocular bioadhesives: Two new avenues for ophthalmic drug delivery. Drug Dev. Ind. Pharm..

[15] Janga K.Y., Tatke A., Balguri S.P., Lamichanne S.P., Ibrahim M.M., Maria D.N., Jablonski M.M., Majumdar S. (2018). Ion-sensitive in situ hydrogels of natamycinbilosomes for enhanced and prolonged ocular pharmacotherapy: In vitro permeability, cytotoxicity and in vivo evaluation. Artif. Cells Nanomed. Biotechnol..

[16] Mundada A.S., Shrikhande B.K. (2006). Design and evaluation of soluble ocular drug insert for controlled release of ciprofloxacin hydrochloride. Drug Dev. Ind. Pharm..

[17] Furqan A.M., Tejal G.S., Dinesh O.S. (2016). A review on therapeutic contact lenses for ocular drug delivery. Drug Deliv..

[18] Willoughby C.E., Batterbury M., Kaye S.B. (2002). Collagen Corneal Shields. Surv. Ophthalmol..

[19] Di Colo G., Burgalassi S., Chetoni P., Fiaschi M.P., Zambito Y., Saettone M.F. (2001). Gel-forming erodible inserts for ocular controlled delivery of ofloxacin. Int. J. Pharm..

[20] Saettone M.F., Salminen L. (1995). Ocular inserts for topical delivery. Adv. Drug Deliv. Rev..

[21] Adelli G.R., Balguri S.P., Bhagav P., Raman V., Majumdar S. (2017). Diclofenac sodium ion exchange resin complex loaded melt cast films for sustained release ocular delivery. Drug Deliv..

[22] Balguri S.P., Adelli G.R., Tatke A., Janga K.Y., Bhagav P., Majumdar S. (2017). Melt-Cast noninvasive ocular inserts for posterior segment drug delivery. J. Pharm. Sci..

[23] Goutham R.A., Tushar H., Punyamurthula N., Balguria S.P., Majumdarabc S. (2015). Evaluation of topical hesperetin matrix film for back-of-the-eye delivery. Eur. J. Pharma. Biopharm..

[24] Di Colo G., Burgalassi S., Chetoni P., Fiaschi M.P., Zambito Y., Saettone M.F. (2001). Relevance of polymer molecular weight to the in vitro/in vivo performances of ocular inserts based on poly(ethylene oxide). Int. J. Pharm..

[25] Anuradha V.G., Chetan P.P., Richard S.G., Melissa G., Kristin P., Ramakrishnan S. (2013). Ophthalmic compositions comprising polyvinyl capralactam-polyvinyl acetate-polyethylene glycol graft copolymers. U.S. Patent.

[26] Majumdar S., Srirangam R. (2009). Solubility, stability, physicochemical characteristics and in vitro ocular tissue permeability of hesperidin: A natural bioflavonoid. Pharm. Res..

[27] Manvi F.V., Patil M.B., Mastiholimath V.S., Rathod R. (2004). Development and evaluation of ocular films of cromolyn sodium. Ind. J. Pharmac. Sci..

[28] Sreenivas S.A., Hiremath S.P., Godbole A.M. (2006). Ofloxacin ocular inserts: Design, formulation and evaluation. Iran. J. Pharmacol. Ther..

[29] Mona H.A., Azza A.M. (2011). Biodegradable ocular inserts for sustained delivery of brimonidine tartarate: Preparation and in vitro/in vivo evaluation. AAPS PharmSciTech.

[30] Marwa S. (2014). Formulation, in vitro and in vivo evaluation of lidocaine HCl ocular inserts for topical ocular anesthesia. Arch. Pharm. Res..

[31] Akshaya T., Narendar D., Karthik Y.J., Sai P.B., Bharathi A., Monica M.J., Soumyajit M. (2019). In situ gel of triamcinolone acetonide-loaded solid lipid nanoparticles for improved topical ocular delivery: Tear kinetics and ocular disposition studies. Nanomaterials.

